# Kentucky State Dental Association

**Published:** 1883-07

**Authors:** 


### PROCEEDINGS.

### Kentucky State Dental Association.

The annual meeting of this body was held June 5th, 6th, and 7th, in the rooms of the Polytechnic Society, Louisville. The meeting was called to order on Tuesday, the 5th, at two o'clock p. m., President J. Hooper in the chair. After the reading of the minutes, and the roll call, the President's address was delivered, which will appear in the August number of the REGISTER. Dr. L. W. Wells, of Paducah, Ky., having arranged to leave the State, sent in his resignation, which was accepted, with regrets at losing so valuable a member.

Dr. Peabody moved that the hours for the sessions of the society should be from 9 to 12 o'clock, and from 2:30 to 6 o'clock. This was approved. The society then adjourned to 9 o'clock tomorrow morning.

WEDNESDAY, SECOND DAY.

The society met according to adjournment, the President in the chair, and a fair number in attendance. The minutes of the society were approved, after which Dr. J. M. Clide, of Covington, read an article on Pathology, which called forth considerable discussion, by Drs. H. A. Smith, A. W. Smith, W. H. Morgan, and R. C. Morgan. Many interesting thoughts and points were presented in the discussion. On motion of Dr. Dunn all visitors and physicians were invited to participate in the discussion.

The committee on chemistry was granted further time for the completion of their report. Adjourned.

AFTERNOON SESSION.

The society convened at 2:30 o'clock with a good attendance.

Prof. J. S. Cassidy, of Covington, read a paper on germs and acids in dental decay. The subject was discussed by Prof. Tobin, Drs. H. A. Smith, H. W. Morgan, J. S. Cassidy and others.

A committee, consisting of Drs. F. Peabody, W. H. Morgan, and A. W. Smith, was appointed to draft appropriate resolutions on the death of W. H. Goddard.

---

A synopsis of the paper appears in this number of the REGISTER.

On motion the association adjourned until 8 o'clock, when Prof. T. W. Tobin lectures on the art of cooking.

EVENING SESSION.

The society convened at 8:30 o'clock, when a large audience was present. The subject was treated in the clear and characteristic style of the Professor, and enlisted the close attention and interest of the audience from the beginning to the end. Quite a number of experiments were made which were very satisfactory. Several kinds of food were cooked by the Professor, in a superior manner, each of which was tested by a delighted audience. The Professor presented the science of cooking in quite a different aspect from that in which it is ordinarily viewed.

The society has decided to hold its annual sessions in Louisville, and to accept the invitation of the Polytechnic Society to meet in their rooms and receive such facilities as can there be granted in the way of apparatus and appliances. Adjourned.

THURSDAY, THIRD DAY.

The association met according to adjournment at 9 o'clock, the President in the chair. A large number was present. Dr. Hooper read an interesting paper on mechanical dentistry which elicited considerable discussion, many of the members taking part.

Dr. H. A. Smith then exhibited a new method of mounting artificial crowns on the roots of teeth, after which the society went into the election of officers for the ensuing year, and the following were chosen:

President.—Dr. Wm. Van Antwerp, Mt. Sterling.

Vice President.—Dr. J. M. Clide, Covington.

Secretary.—Dr. C. E. Dunn, Louisville.

Treasurer.—Dr. J. F. Canine, Louisville.

The following delegates were appointed to the American Dental Association:

Drs. C. E. Canine, Garnett, J. F. Canine, A. W. Smith, and E. S. Buckner.

The following were appointed:

Executive Board, C. G. Edwards; Board of Censors, E. S. Buckner; and Board of Examiners, Dr. J. S. Cassidy.

The society next passed a resolution thanking Prof. Tobin and the Polytechnic Society for services rendered; and adjourned to meet in this city on the first Tuesday in June, 1884.

